# Differences between Adiposity Indicators for Predicting All-Cause Mortality in a Representative Sample of United States Non-Elderly Adults

**DOI:** 10.1371/journal.pone.0050428

**Published:** 2012-11-30

**Authors:** Henry S. Kahn, Kai McKeever Bullard, Lawrence E. Barker, Giuseppina Imperatore

**Affiliations:** Division of Diabetes Translation, U. S. Centers for Disease Control and Prevention, Atlanta, Georgia, United States of America; University of Texas Health Science Center at San Antonio, United States of America

## Abstract

**Background:**

Adiposity predicts health outcomes, but this relationship could depend on population characteristics and adiposity indicator employed. In a representative sample of 11,437 US adults (National Health and Nutrition Examination Survey, 1988–1994, ages 18–64) we estimated associations with all-cause mortality for body mass index (BMI) and four abdominal adiposity indicators (waist circumference [WC], waist-to-height ratio [WHtR], waist-to-hip ratio [WHR], and waist-to-thigh ratio [WTR]). In a fasting subsample we considered the lipid accumulation product (LAP; [WC enlargement*triglycerides]).

**Methods and Findings:**

For each adiposity indicator we estimated linear and categorical mortality risks using sex-specific, proportional-hazards models adjusted for age, black ancestry, tobacco exposure, and socioeconomic position. There were 1,081 deaths through 2006. Using linear models we found little difference among indicators (adjusted hazard ratios [aHRs] per SD increase 1.2–1.4 for men, 1.3–1.5 for women). Using categorical models, men in adiposity midrange (quartiles 2+3; compared to quartile 1) were not at significantly increased risk (aHRs<1.1) unless assessed by WTR (aHR 1.4 [95%CI 1.0–1.9]). Women in adiposity midrange, however, tended toward elevated risk (aHRs 1.2–1.5), except for black women assessed by BMI, WC or WHtR (aHRs 0.7–0.8). Men or women in adiposity quartile 4 (compared to midrange) were generally at risk (aHRs>1.1), especially black men assessed by WTR (aHR 1.9 [1.4–2.6]) and black women by LAP (aHR 2.2 [1.4–3.5]). Quartile 4 of WC or WHtR carried no significant risk for diabetic persons (aHRs 0.7–1.1), but elevated risks for those without diabetes (aHRs>1.5). For both sexes, quartile 4 of LAP carried increased risks for tobacco-exposed persons (aHRs>1.6) but not for non-exposed (aHRs<1.0).

**Conclusions:**

Predictions of mortality risk associated with top-quartile adiposity vary with the indicator used, sex, ancestry, and other characteristics. Interpretations of adiposity should consider how variation in the physiology and expandability of regional adipose-tissue depots impacts health.

## Introduction

The best clinical measures of adiposity for predicting future health risks are not clear. Ascending categories of body mass index (BMI, kg/m^2^) generally define increasing degrees of adiposity [1], but this widely employed indicator cannot account for the weight contributions made by different organs, lean and fat tissues, or the physiology of body-fat distribution [2]. A recent Scientific Statement from the American Heart Association acknowledged substantial heterogeneity in adult body fatness at a given BMI, but it also recognized assessment opportunities related to body-fat distribution and ectopic fat deposition [3]. The review’s authors endorsed the use both of BMI measurements (at cutpoints 25, 30, 35, and 40) and of waist circumference (WC) as tools for assessing health risk associated with adiposity. They drew attention, however, to an absence in the literature of established WC cutpoints that would be specific to BMI level, sex, age, or ancestral groups.

We have explored how the BMI, WC, and four other adiposity indicators were associated with the all-cause mortality experienced by a representative sample of US non-elderly adults. In conventional, sex-stratified analyses we evaluated each adiposity indicator linearly as a continuous variable. Since we could not assume the existence of linear mortality relationships, we also evaluated each indicator as a categorical variable defined by comparing subgroups of adults (ordinal quartiles) defined by the boundary of each indicator’s 25^th^ percentile (p25) or the boundary of its 75^th^ percentile (p75) in the sex-stratified, overall population. By evaluating all six adiposity indicators in this manner, we hoped to identify differences in mortality prediction by these indicators when applied to non-elderly men, women, and population subgroups defined by various characteristics.

## Population and Methods

### Study Population

Our baseline sample came from participants in the third National Health and Nutrition Examination Survey (NHANES III), a complex multistage, clustered, stratified probability sample of the US civilian, noninstitutionalized population in 1988–1994 [4]. The analytic cohort included adults who were aged 18–64 years, not pregnant, had complete anthropometric data, and had no history of cancer (with the exception of nonmelanoma skin cancer). We included eligible persons whose self-identified race and ethnicity placed them in one of three categories: non-Hispanic black, Mexican-American (both oversampled in NHANES III), or non-Hispanic white. In this paper we hereafter refer to these categories as “ancestries” [5] to acknowledge the complex contributions of historical, sociocultural, and biological factors. We excluded persons who identified themselves by other races or ethnicities due to their small numbers and heterogeneous descriptions. We also excluded persons who were ineligible for mortality ascertainment (0.1% of sample) because of insufficient personal identifying information [6]. Our remaining analytic cohort contained 5,514 men and 5,923 women.

### Baseline Variables

Participants completed a household interview and an examination with standardized measurements of weight, height, standing WC (in the horizontal plane at the level just above the iliac crest, at minimal respiration), standing hip circumference (at the maximum extension of the buttocks), and midthigh circumference (in seated position at the midpoint of the right thigh) [7]. Height and circumferences were reported to the nearest 0.1 cm. From these measurements we calculated each participant’s BMI, waist-to-height ratio (WHtR), waist-to-hip ratio (WHR), and waist-to-thigh ratio (WTR).

From a subset of the analytic cohort whose serum had been assayed for fasting triglyceride concentration (n = 6,890, fast durations 8–24 hours) we calculated an additional index, the lipid accumulation product (LAP). LAP is a non-conventional adiposity indicator for adults that incorporates an anthropometric estimate of central adiposity and a laboratory assay of circulating lipid fuels in order to extend the physiological concept of lipid excess [8]. Earlier literature has described several phenotypes of the “hypertriglyceridemic waist,” each defined as a dichotomous indicator [9], [10]. The LAP indicator extends this concept in the form of a continuous variable. Increased values of LAP have been associated with prevalent type 2 diabetes [11]–[13], incident type 2 diabetes [12], [14], hepatic steatosis [15], and insulin resistance [16]. For this calculation we used the formulas:
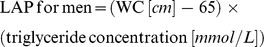


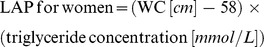



In addition to considering age and three ancestral groups, we adjusted for baseline low socioeconomic position and tobacco exposure since these factors contribute substantially to variation in both adiposity and mortality. For dichotomous indicators of socioeconomic position, we considered both the household poverty-income ratio and the self-reported educational attainment [17], [18]. The poverty-income ratio was determined from household interview questions, and missing poverty-income information (8.5% of analytic cohort) was imputed using 5 imputation files prepared by the National Center for Health Statistics [19]. We dichotomized the poverty-income ratio at less than 200% of poverty, a threshold consistent with recent mortality analyses for the US [17], [20], and we dichotomized attained education at less than high school completion [17], [18]. As sole adjustments for low socioeconomic position, our men’s models included only the poverty-income ratio marker and our women’s models included only the high-school completion marker. As shown in Table 1, these choices reflected the sex-specific, relative strengths of these alternative risk factors for mortality.

**Table 1:** Selected baseline characteristics of U.S. adults aged 18–64 years, NHANES 1988–1994.

Characteristic			Total	Alive			Deceased	
					Men	Women	Men	Women
Sample size, n a			11,437		4,858	5,498	656	425
Population estimate, n b			128.3		59.0	59.9	5.6	3.9
Follow-up interval, mean y (SE)			14.7 (0.2)		15.2 (0.2)	15.1 (0.3)	9.2 (0.4)	9.6 (0.3)
Age, % (SE)	18–29 y		29.2 (0.9)		31.4 (1.1)	30.0 (1.1)	12.6 (2.5)	7.9 (2.5)
	30–44 y		40.2 (1.0)		42.6 (1.2)	41.2 (1.3)	20.9 (2.9)	18.7 (3.2)
	45–64 y		30.6 (1.0)		26.0 (1.2)	28.9 (1.2)	66.5 (3.3)	73.4 (3.7)
Age, mean y (SE)			38.1 (0.3)		36.7 (0.3)	37.6 (0.3)	47.7 (0.8)	51.0 (1.0)
Ancestry, % (SE)	Non-Hispanic white		81.0 (0.9)		81.9 (1.0)	80.7 (1.1)	76.8 (2.3)	78.7 (2.2)
	Non-Hispanic black		12.6 (0.8)		11.1 (0.7)	13.5 (0.9)	17.3 (2.0)	17.0 (2.0)
	Mexican-American		6.3 (0.6)		7.0 (0.7)	5.8 (0.5)	5.9 (0.8)	4.3 (0.8)
< HS education, % (SE)			20.2 (1.0)		20.3 (1.0)	17.6 (1.1)	34.9 (3.1)	38.3 (3.2)
<200% poverty ratio, % (SE)			31.4 (1.1)		28.6 (1.1)	32.7 (1.4)	40.4 (2.6)	41.9 (3.5)
Tobacco exposure, % (SE)			36.1 (1.0)		39.5 (1.0)	29.8 (1.1)	56.3 (3.2)	52.4 (2.5)
Prevalent diabetes, % (SE)			4.6 (0.3)		3.7 (0.4)	3.5 (0.4)	16.8 (1.6)	16.7 (1.9)

NHANES = National Health and Nutrition Examination Survey, SE = standard error, HS = high school.

a Unweighted.

b In millions.

Dichotomous active tobacco exposure was inferred for participants whose serum cotinine assay was >10 ng/ml [21], [22]. For participants with missing cotinine assays (5.1% of cohort) we imputed tobacco exposure from variables (including self-reported smoking history) contained in the 5 imputation files.

In our primary, multiply-adjusted models we included no terms for physiologic risk markers at baseline (e.g., diabetes, hypercholesterolemia, hypertension, inflammatory cytokines) because these characteristics can evolve or fluctuate more rapidly than adiposity, their relation to mortality may represent a downstream consequence of increased adiposity, or their inclusion may depend on uncommon laboratory assays. However, we conducted one sub-analysis in which diabetes baseline status was included so that we could determine if results varied by diabetes status. We defined baseline diabetes from self-reports or a concentration of glycated hemoglobin (HbA_1c_)≥6.5% [23].

### Ascertainment of Deaths

The mortality status of the NHANES III participants was ascertained through probabilistic record matching with the National Death Index, a centralized database of all US deaths [6]. Of the original 11,437 eligible cohort members, 1,081 (9.5%) were determined to have died by 31 December 2006. We computed the survival time for each deceased participant from the exact dates of the NHANES III exam and of death from the restricted-use, linked mortality files of the National Center for Health Statistics. Those not deemed to have died by the end of 2006 were treated as alive for these analyses.

### Statistical Analysis

Sampling weights from the NHANES III examinations were used with SAS programs (Release 9.2.2, SAS Institute, Cary, NC) and SUDAAN (Release 10.0.1, Research Triangle Institute, Research Triangle, NC) to estimate the size and characteristics of the represented US non-elderly adult population and the sex-specific, statistical distributions of the six adiposity indicators. The sampling weights employed in SUDAAN accounted for the NHANES III unequal selection probabilities (clustered design, planned oversampling, and differential nonresponse) [24]. For each adiposity indicator, we defined a sex-specific midrange to include those persons in quartiles 2 plus 3 (half of the described population) whose adiposity put them between the indicator’s p25 and p75.

We used PROC SURVIVAL (SUDAAN) to fit Cox proportional-hazard models that estimated each adiposity indicator’s associations with time from baseline examination to death. Sex-specific models evaluated:

1. a linear association with the standardized adiposity indicator (per 1 SD of the continuous variable);
2. a categorical mortality risk associated with being above adiposity boundary p25 (midrange compared with quartile 1 adiposity, ignoring the remote quartile 4); and
3. a categorical mortality risk associated with being above adiposity boundary p75 (quartile 4 adiposity compared with midrange, ignoring the remote quartile 1).

For (1) we used log-transformations of BMI, WC, WHtR, WTR and LAP to bring these variables closer to a normal distribution; log-transformation was not necessary for WHR. In all models we considered results with p<0.05 significant.

To estimate p25 and p75 for each adiposity indicator we first assessed the empirical effects of baseline age by fitting sex-specific cubic models (function of age, age^2^, and age^3^). Cubic models were considered because they are a flexible family of non-linear curves. The value of all the indicators rose with increased age up to about 45 years old, but, at older ages, the men’s age relationship was generally more curvilinear (inverted U shape) than that observed for the women. We therefore estimated p25 and p75 for four subpopulations represented by the cross-class of sex and age groups 18–44 or 45–64 years.

These linear and categorical associations are reported as sex-specific hazard ratios (HRs) either unadjusted or multiply adjusted for age, ancestry and specified dichotomous variables. Because some of the adjusted Cox models for men included a significant term for age^2^, we, to maintain consistency, included an age^2^ term for all men’s adjusted models (but not for women’s adjusted models). With inclusion of three categories of ancestry in our models we identified interactions of adiposity with non-Hispanic black ancestry (compared to non-Hispanic whites) but not with Mexican Americans; we therefore collapsed non-Hispanic white and Mexican American into one category because there was little difference between them.

## Results

Our analytic sample represented a US population of 128 million non-elderly adults with a baseline mean age of 38.1 years (Table 1). During the follow-up period (up to 18.1 years), deaths occurred among an estimated 8.7% of the men (baseline mean age of 47.7 years) and 6.1% of the women (baseline mean age of 51.0 years). Deaths were more likely among non-Hispanic blacks, those with education less than high school (especially women), those with income below 200% of the poverty threshold (especially men), and those with baseline tobacco exposure.

At baseline, irrespective of sex and the adiposity indicator used, the older participants (ages 45–64) had greater adiposity than the younger participants (Table 2). At p25, p50 and p75 the men had higher adiposity values than women for WC, WHR, WTR and LAP, but this sex difference was not consistently seen for BMI or WHtR.

**Table 2:** Median (*p50*) and interquartile boundary values (p25, p75) for baseline adiposity indicators by age group and sex among U.S. adults, 1988–1994.

Adiposity indicator	Total			18–44 years						45–64 years					
				Men			Women			Men			Women		
	p25	*p50*	p75	p25	*p50*	p75	p25	*p50*	p75	p25	*p50*	p75	p25	*p50*	p75
BMI, *kg/m^2^*	22.4	25.3	29.2	22.8	25.2	28.2	21.0	23.6	28.4	24.3	26.7	29.9	23.4	26.8	31.5
WC, *cm*	79.2	89.5	100.0	82.1	90.4	99.3	73.0	80.7	91.6	92.0	98.5	106.3	83.0	92.3	103.3
WHtR	0.47	0.52	0.58	0.47	0.51	0.56	0.45	0.49	0.56	0.52	0.56	0.61	0.51	0.57	0.64
WHR	0.83	0.89	0.95	0.87	0.91	0.96	0.77	0.82	0.87	0.94	0.98	1.03	0.83	0.88	0.94
WTR	1.60	1.72	1.87	1.63	1.73	1.84	1.50	1.61	1.72	1.84	1.93	2.04	1.66	1.79	1.95
LAP^a^, *cm·mmol/L*	15.9	30.3	60.1	15.6	28.8	56.6	11.5	20.8	38.4	30.2	50.2	84.9	25.3	47.6	82.8

a LAP = lipid accumulation product (estimates derived from fasting participants; n = 6,890)

Population-based cross-tabulations demonstrated that, when comparing any two adiposity indicators, substantial portions of US non-elderly adults had discordant assignments to quartile 1, midrange, or quartile 4 (see supplementary appendix).

### Unadjusted Mortality Prediction

In our unadjusted models each linear adiposity indicator was positively associated with mortality. The hazard ratios (HRs, per 1 SD adiposity increment) ranged from 1.3 [95%CI 1.2–1.5] (men’s BMI) to HR 2.4 [2.1–2.8] (women’s WTR). WTR was a stronger linear predictor than other adiposity indicators among both men (Table 3) and women (Table 4).

**Table 3:** Hazard ratios (95% CI) for all-cause mortality associated with 6 adiposity indicators presented as linear continuous models and categorical models at boundaries p25 or p75 for US nonelderly men.

Indicator	Unadjusted models			Multiply adjusted models^a^		
	Linear HRs	Categorical HRs		Linear aHRs	Categorical aHRs	
	*Continuous (per SD)*	*At p25* b	*At p75* c	*Continuous (per SD)*	*At p25* b	*At p75* c
BMI	1.32 (1.15–1.51)	0.78 (0.57–1.06)	1.50 (1.16–1.94)	1.24 (1.06–1.45)	0.78 (0.56–1.09)	1.54 (1.18–2.01)
WC	1.52 (1.32–1.75)	0.83 (0.62–1.10)	1.62 (1.23–2.14)	1.27 (1.08–1.51)	0.85 (0.63–1.15)	1.54 (1.18–2.02)
WHtR	1.62 (1.39–1.88)	0.97 (0.68–1.38)	1.79 (1.35–2.36)	1.33 (1.11–1.59)	0.91 (0.63–1.31)	1.70 (1.31–2.19)
WHR	1.71 (1.51–1.94)	1.17 (0.87–1.58)	1.19 (0.92–1.53)	1.27 (1.09–1.48)	1.03 (0.75–1.42)	1.23 (0.94–1.61)
WTR	2.11 (1.78–2.49)	1.51 (1.09–2.09)	1.38 (1.03–1.87)	1.43 (1.20–1.71)	1.38 (0.98–1.93)	1.13 (0.86–1.49)
LAP	1.49 (1.21–1.83)	1.03 (0.74–1.45)	1.10 (0.64–1.89)	1.22 (0.95–1.55)	1.03 (0.72–1.49)	1.11 (0.66–1.85)
P-value d	<0.001	0.042	0.25	0.86	0.22	0.18

HR = hazard ratio, aHR = multiply adjusted hazard ratio.

a Models for **men** were adjusted for age, age^2^, black ancestry, tobacco exposure, and income <200% of poverty threshold.

b Risk comparing midrange *vs* quartile 1,

c Risk comparing quartile 4 *vs* midrange.

d P-values determined from chi-squared test evaluating 6 adiposity indicators (5 degrees of freedom).

**Table 4:** Hazard ratios (95% CI) for all-cause mortality associated with 6 adiposity indicators presented as linear continuous models and categorical models at boundaries p25 or p75 for US nonelderly women.

Indicator	Unadjusted models			Multiply adjusted models^a^		
	Linear HRs	Categorical HRs		Linear aHRs	Categorical aHRs	
	*Continuous (per SD)*	*At p25* b	*At p75* c	*Continuous (per SD)*	*At p25* b	*At p75* c
BMI	1.50 (1.39–1.62)	1.30 (0.91–1.85)	1.42 (1.08–1.86)	1.32 (1.19–1.47)	1.24 (0.87–1.77)	1.54 (1.18–2.00)
WC	1.86 (1.71–2.03)	1.85 (1.37–2.49)	1.65 (1.31–2.08)	1.47 (1.29–1.67)	1.50 (1.06–2.13)	1.64 (1.30–2.07)
WHtR	1.88 (1.73–2.05)	1.83 (1.26–2.66)	1.63 (1.24–2.14)	1.45 (1.29–1.64)	1.39 (0.98–1.99)	1.65 (1.26–2.17)
WHR	1.43 (1.25–1.63)	1.90 (1.08–3.34)	2.33 (1.81–3.00)	1.30 (1.17–1.46)	1.23 (0.71–2.14)	1.80 (1.42–2.27)
WTR	2.41 (2.09–2.77)	1.73 (1.10–2.72)	2.23 (1.69–2.93)	1.53 (1.31–1.78)	1.20 (0.79–1.82)	1.72 (1.28–2.31)
LAP	1.80 (1.55–2.08)	1.89 (1.11–3.20)	1.53 (0.95–2.46)	1.27 (1.02–1.57)	1.26 (0.75–2.15)	1.48 (0.90–2.43)
P-value d	<0.001	0.70	0.053	0.35	0.96	0.95

HR = hazard ratio, aHR = multiply adjusted hazard ratio.

a Models for **women** were adjusted for age, black ancestry, tobacco exposure, and education<high school graduation.

b Risk comparing midrange *vs* quartile 1,

c Risk comparing quartile 4 *vs* midrange.

d P-values determined from chi-squared test evaluating 6 adiposity indicators (5 degrees of freedom).

Evaluated categorically, WTR was the only indicator that significantly predicted mortality for both sexes at the p25 boundary (midrange *vs* quartile 1, HRs 1.5–1.7) and the p75 boundary (quartile 4 *vs* midrange, HRs 1.4–2.2).

### Multiply Adjusted Mortality Prediction

The linear associations with mortality were attenuated in models adjusted for age, black ancestry, tobacco exposure and socioeconomic position (Tables 3 and 4). In these adjusted models we found little variation in linear risk among indicators (adjusted hazard ratios [aHRs] 1.2–1.4 for men, 1.3–1.5 for women). For both sexes WTR was marginally stronger than the other continuous indicators.

**Figure 1:**
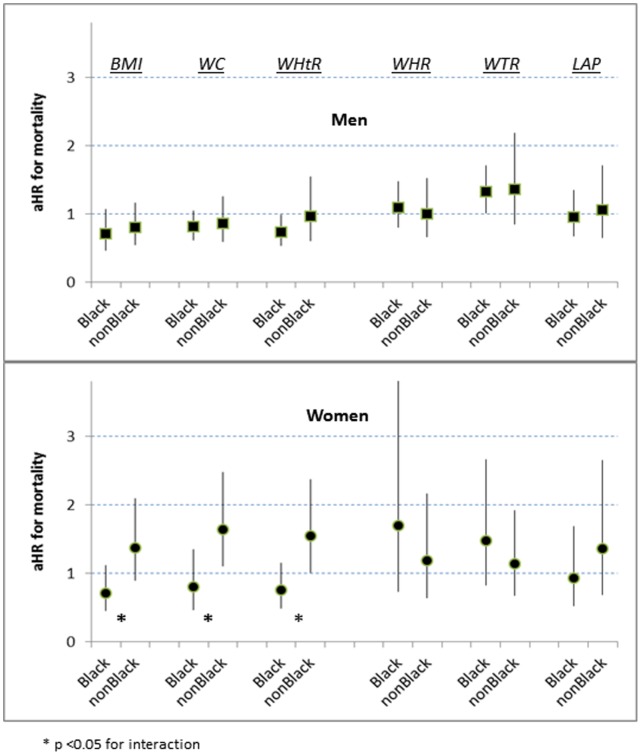
Interactions with ancestral group for mortality risk at p25, by 6 adiposity indicators. (aHR = multiply adjusted hazard ratio).

At the p25 boundary five of the men’s adiposity indicators had no significant categorical association with increased mortality (aHRs 0.8–1.0), but WTR showed a modest increased risk (aHR 1.4 [1.0–1.9]) (Table 3 and Figure 1). For women overall at the p25 boundary the mortality risks were similar for each indicator (aHRs 1.2–1.5) (Table 4). However, when black women were assessed by BMI, WC, or WHtR they were not at significantly increased risk (aHRs 0.7–0.8 vs 1.4–1.6 for non-Black women) (Figure 1).

**Figure 2:**
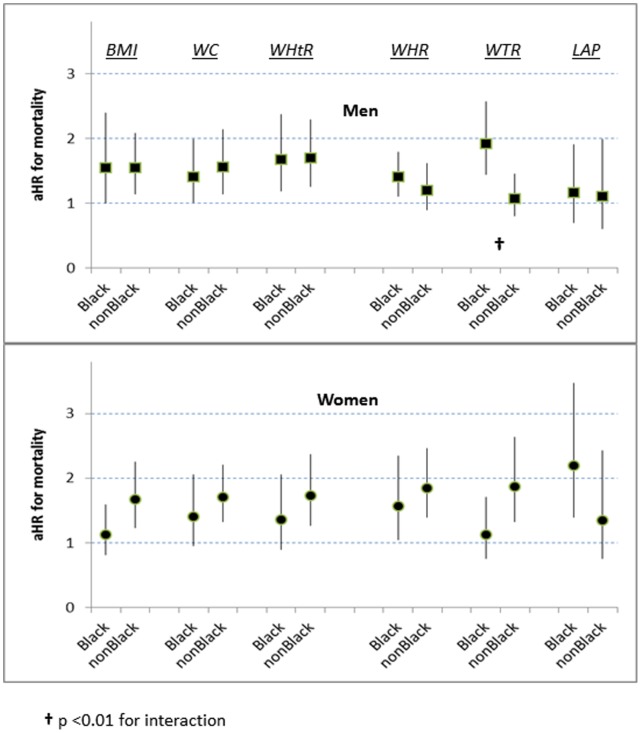
Interactions with ancestral group for mortality risk at p75, by 6 adiposity indicators. (aHR = multiply adjusted hazard ratio).

**Figure 3:**
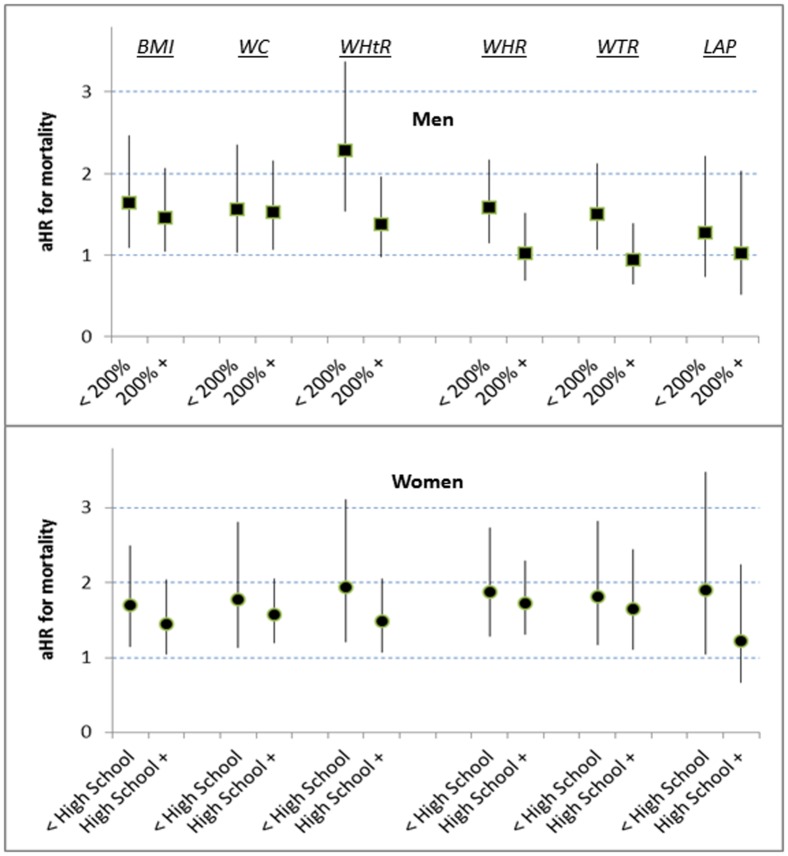
Interactions with socioeconomic position (poverty-income ratio or high-school completion) for mortality risk at p75, by 6 adiposity indicators. (aHR = multiply adjusted hazard ratio).

At the p75 boundary the significant associations of BMI, WC, and WHtR with mortality were similar for men and women (aHRs 1.5–1.7) (Tables 3 and 4), and we found no interactions between these indicators and ancestral group (Figure 2). However, for low-income men, compared to men with higher income, quartile 4 of WHtR appeared to have a greater increased risk (aHR 2.3 [1.5–3.4]) (Figure 3; p = 0.07 for interaction).

With assessment by WHR at adiposity p75 the increased mortality risk for men overall was weak (aHR 1.2 [0.9–1.6]) (Table 3), but for low-income men the risk by WHR was possibly increased (aHR 1.6 [1.2–2.2]) (Figure 3; p = 0.085 for interaction). For women overall at p75 WHR was strongly associated with risk (Table 4) irrespective of ancestry (Figure 2) or socioeconomic position (Figure 3).

Assessment by WTR at p75 for men overall was not significantly associated with mortality risk (Table 3). For black men, however, WTR at p75 was strongly associated with mortality (aHR 1.9 [1.4–2.6]) (Figure 2), and low-income men also had an increased risk (aHR 1.5 [1.1–2.1]) (Figure 3; p = 0.08 for interaction) For women overall, WTR at p75 was associated with substantial mortality risk (aHR 1.7 [1.3–2.3]) (Table 4), but this risk estimated by WTR was much less for black women (aHR 1.1 [0.8–1.7]) (Figure 2; p = 0.07 for interaction). By contrast, black women assessed by LAP at p75 had a high mortality risk (aHR 2.2 [1.4–3.5]) (Figure 2).

**Figure 4:**
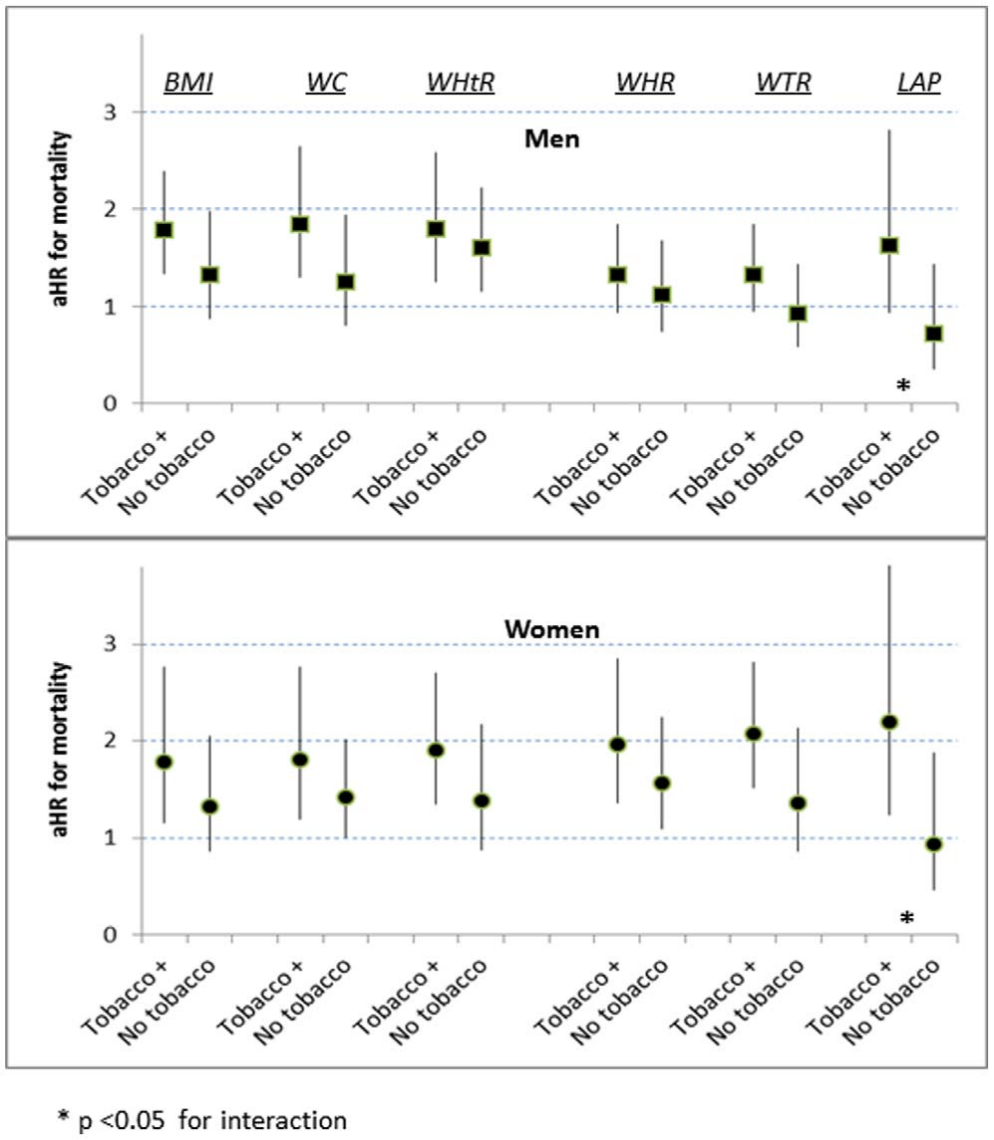
Interactions with tobacco exposure for mortality risk at p75, by 6 adiposity indicators. (aHR multiply adjusted hazard ratio).

At p75, for both sexes and all indicators, higher adiposity was associated with greater mortality risks among persons with tobacco exposure than without (Figure 4). The interactions with tobacco exposure were only significant for adiposity assessed by LAP.

In all categorical models with multiple adjustments there were substantial mortality risks associated with tobacco exposure (aHRs 1.9–3.3), men’s poverty status (aHRs 1.7–1.8) and women’s low educational attainment (aHRs 1.5–1.7). The aHRs for these binary risk factors were generally larger than those associated with terms for categorical adiposity at either p25 or p75 (data not shown).

### Differences Related to Baseline Diabetes Status or Age Group

**Figure 5:**
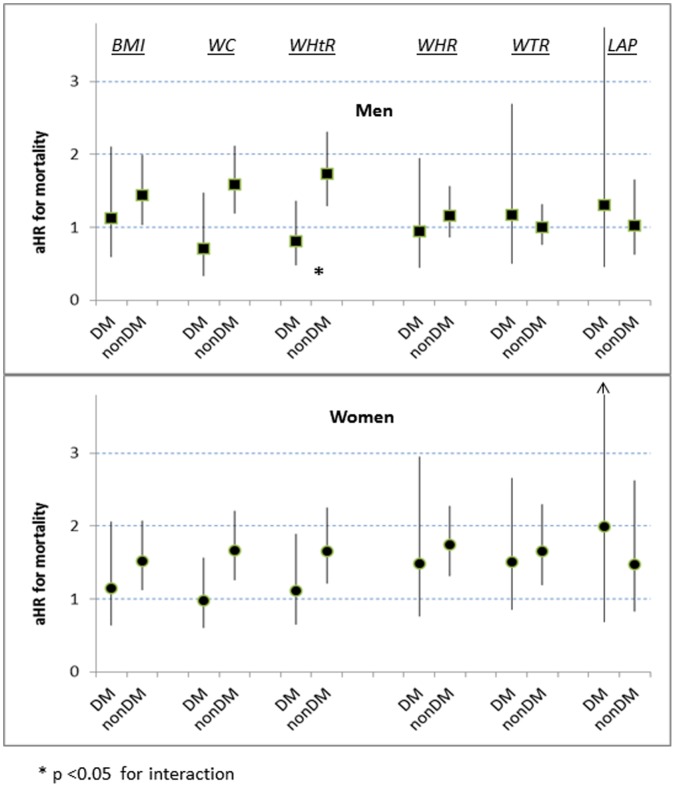
Interactions with baseline diabetes for mortality risk at p75, by 6 adiposity indicators. (aHR = multiply adjusted hazard ratio).

In expanded categorical models that included a term for baseline diabetes status, we found that WC and WHtR at the p75 boundary were significantly associated with increased mortality for non-diabetic men and women (aHRs 1.6–1.7), but not for those who had diabetes (aHRs 0.7–1.1) (Figure 5). At p25 we identified no differences in mortality risk by baseline diabetes and any of the adiposity indicators.

In expanded models with a term for baseline age groups 18–44 *vs* 45–64 years (but retaining also continuous terms for age) we found no age-group interaction for either sex at p25 or for women at p75. However, WTR for men at p75 was unrelated to mortality for the younger age group (aHR 1.0 [0.8–1.4]), but it carried a high risk for men ages 45–64 (aHR 2.4 [1.5–3.8]) (not shown in a figure; p = 0.002 for interaction).

## Discussion

Previous prospective studies of community-based, non-elderly adults have described how all-cause mortality was associated with measured BMI [25]–[35], WC [26]–[30], [33], [34], [36], [37], WHtR [26], [33], [34], WHR [26]–[28], [30], [33], [34], [36], [38] or WTR [29], [30]. Most of the cohorts occurred in Europe, Asia or Australia. One report was a large meta-regression in which 27% of the participants came from the US, but BMI was the only indicator analyzed in that study [31]. The other cited articles with US participants were earlier analyses of participants in the NHANES III examination [25], [30]; our report from the same baseline population benefits from six additional years of mortality experience.

We believe ours is the first analysis of nationally representative data to include all-cause mortality estimates associated with these five conventional adiposity indicators, and we have also included mortality estimates associated with LAP. Some of these six indicators had distinctly non-linear associations with all-cause mortality. Therefore, in order to simplify the comparison of all six indicators, we have reported categorical hazard ratios that predicted mortality for persons in each indicator’s midrange (compared to those below p25) and for persons above the indicator’s p75 (compared to those in the midrange).

Among the continuous, unadjusted adiposity indicators WTR had the strongest association with mortality, and this ranking was preserved in the multiply adjusted models. These results mirror an earlier prospective analysis of WTR and mortality in the Canadian Fitness Survey [29]. Despite different anthropometric protocols, both the Canadian Fitness Survey and our NHANES analysis demonstrated that information on thigh size relative to waist size can enhance mortality prediction. These enhancements in risk estimation depended, however, on sex and whether our categorical analysis was made at adiposity boundary p25 or p75. Among men at p25, an increase in waist size alone was not significantly associated with increased mortality, but the incorporation of information about thigh size (in the denominator of WTR) substantially increased their estimated risk (Table 3 and Figure 1). We infer that the men’s increased mortality risk in the WTR midrange is not related primarily to an expanded WC but to a relatively diminished thigh circumference. As a corollary inference, men in quartile 1 of WTR are protected against mortality by their large thighs relative to their waists.

Thigh expansion among men is less common than among women, but for both sexes an increase in thigh size has been associated with reduced all-cause mortality [29], [39]. In contrast to upper-body adipose tissue, the volume of fat in the lower body tends to be less responsive to short-term variations in nutrient intake [40]. The existence of metabolic benefits associated with large thighs is supported by cross-sectional studies of non-elderly adults that demonstrated larger leg-fat mass was associated with lower levels of circulating triglycerides and total cholesterol/HDL cholesterol, and higher levels of HDL-cholesterol and insulin sensitivity [41], [42]. Other benefits of an enlarged subcutaneous depot of gluteofemoral adipose tissue may include decreased circulating inflammatory cytokines, increased adiponectin, and an enhanced capacity of lower-body adipocytes to buffer or sequester fatty acids that would otherwise contribute to harmful deposition of lipid metabolites in ectopic (non-adipose) tissues [43]–[45].

Adults above the p75 boundary of WTR might include many persons with limited expandability of the adipose tissue in their thighs. We note with interest that black men in quartile 4 of WTR have markedly increased mortality risk, but this adverse association was not found for black women in quartile 4 (Figure 2).

The reduced mortality observed among men with BMI, WC, or WHtR in the midrange relative to quartile 1 (Table 3) is consistent with the previous international literature showing a J-shaped risk curve for both sexes at the lower values of these three adiposity indicators [27], [28], [30], [31], [34]–[37]. Contrary to expectation, the women in our cohort tended to have increased mortality risk in adiposity midrange assessed by BMI, WC, or WHtR (Table 4), but these increased risk estimates above p25 applied specifically to non-Black women (Figure 1). It is possible that some US women examined in NHANES III – primarily those non-Black – were different from women with baseline body measurements described in earlier cohorts or from other countries. The US Cancer Prevention Study II that began a decade before NHANES III reported little difference in all-cause mortality experience between white women with baseline BMI <22 kg/m^2^ (comparable to our BMI quartile 1) and those with BMI 22.0 to 29.9 (comparable to our BMI midrange) [46]. This large, often cited cohort, however, depended on self-reported weight and height, had no objective indicator of tobacco use, excluded participants <30 years old, and underrepresented persons with low educational level. Non-elderly women in BMI quartile 1 from other environments possibly shared unfavorable nutritional or social circumstances that were associated with an increased mortality risk. By contrast, non-Black women in BMI quartile 1 from our US cohort may have included many who benefited from substantial social privilege despite having low levels of adiposity. A recent cohort reported from Mauritius has also found that women in WC quartile 1 had reduced risk of mortality if they were of South Asian ancestry (absent J-shape) but increased mortality risk if they were of African ancestry [37]. Mauritian men in WC quartile 1 of either ancestry had comparably increased mortality risk.

Conventional assessments of increased adult adiposity depend on a BMI threshold value ≥30 kg/m^2^ (“obesity”) irrespective of age, sex or clinical circumstances [1], [3]. This threshold is close to the BMI value for p75 in our NHANES III sample of non-elderly US adults (Table 2). At this upper boundary of adiposity midrange the BMI associations with all-cause mortality (aHR 1.5 for men or women) suggest that adiposity’s impact on long-term health could have been assessed at least as well by the WC or WHtR (Tables 3 and 4). However, despite categorical risk estimates that were similar for BMI, WC, and WHtR at p75, the individuals located in the midrange or quartile 4 were frequently different depending on the indicator used (see the estimated prevalences of discordance in the supplementary appendix). More recent surveys of non-elderly adults in Finland suggest that discordances between these 3 adiposity indicators may have increased since about 1997 [47].

Given that “obese” individuals located in quartile 4 of BMI might be located in the midrange of an abdominal adiposity indicator (or *vice versa*), our mortality predictions at p75 for BMI could be interpreted in conjunction with mortality predictions at the p75 values for an alternative adiposity indicator. The availability of population-based p75 values of WC or WHtR, for example, begins to respond to the American Heart Association’s request for abdominal adiposity cutoff values specific to BMI, age, and sex [3].

Among adults aged 18–44 years with a BMI of ∼30 kg/m^2^ a supplementary health-risk estimate might depend on a men’s WC p75 threshold value of ∼99 cm and women’s WC p75 threshold value of ∼92 cm (Table 2). For adults 45–64 years old, a supplementary health-risk estimate might depend on a men’s WC p75 threshold of ∼106 cm and women’s WC p75 threshold of ∼103 cm. For black women, a BMI value above 30 is associated with only a weakly increased risk (aHR 1.1 [0.8–1.6]; Figure 2). Indeed, contemporary estimates from other sources suggest that the cardiometabolic risk [48] and mortality risk [49] for US black women begin to rise significantly only above ∼33 kg/m^2^. Assessing black women’s risk by the p75 WC threshold instead of the BMI threshold might better clarify their true risk. An alternative assessment for black women using the p75 threshold value for LAP instead of BMI could provide a substantially higher risk estimate (Figure 2). For persons of ancestries other than non-Hispanic white, non-Hispanic black, or Mexican Americans we cannot be certain whether different adiposity thresholds would be better markers of health risk.

Similar to supplementary assessments using WC, the WHtR could likewise provide refined risk estimates for persons with BMI ∼30 kg/m^2^. Since the p75 for WHtR ranges from ∼0.56 to ∼0.64 for all non-elderly adults (Table 2), a practical, simplified estimate of health risk among persons with BMI ∼30 kg/m^2^ could depend on rounding the WHtR p75 threshold value to 0.60 irrespective of sex and age. The same “pragmatic” WHtR threshold value has recently been proposed to identify a “Take Action” adiposity level associated with increased health risk [50].

Among men and women with diabetes at baseline we found that neither WC nor WHtR above p75 was associated with increased mortality risk (Figure 5). Recent, large, observational studies including older participants have described a similar “obesity paradox” in which the diabetic adults with BMI ≥30, compared to the diabetic persons in lower BMI categories, had mortality risks that were reduced [51], [52] or similar [53]. Compared to the risk for non-diabetic adults, the attenuation of relative risk in quartile 4 may occur because diabetes itself already carries an increased risk of mortality, and thus adiposity contributes very little further detriment to health. However, risk estimation for diabetic patients using thresholds of LAP at p75 would provide higher relative-risk estimates than those provided by WC or WHtR. Use of WTR thresholds would also yield a similarly increased relative-risk estimate, possibly because reduced thigh size is associated with an increase in circulating triglycerides [41]. These advantages of LAP and WTR raise interesting issues about the pathophysiological consequences for diabetic patients of having an increased concentration of circulating triglycerides. An older cohort of high-risk patients from the US found that LAP predicted mortality better among persons without diabetes [54], whereas one from Germany found that LAP predicted mortality better among patients with diabetes [13]. Our community-based US cohort cannot resolve this conflict regarding baseline diabetes status in clinic-derived cohorts.

The weak association between LAP and all-cause mortality in our cohort overall is compatible with recent reports from smaller cohorts of older adults [13], [55]. However, at the p75 boundary increased LAP predicted mortality relatively well among persons with tobacco exposure (Figure 4). Among heavy smokers the concentration of circulating triglycerides is increased [56], [57], and, since the definition of LAP extends the concept of lipid overload by including a laboratory assay of circulating triglycerides, the value of the LAP expression is closely tied to hypertriglyceridemia. Quartile 4 of LAP, therefore, likely includes an excess of heavy smokers. The increased mortality for tobacco-exposed persons above LAP p75 probably reflects their risk linked to smoking levels beyond what was captured by our binary adjustment for tobacco exposure.

The limited ability of LAP to predict mortality might be explained to some degree by LAP’s association with hepatic steatosis [15]. A recent analysis of over 11,000 adult NHANES III participants reported that fatty liver (as assessed from ultrasound images of the gallbladder) had no association with increased mortality [58]. This unexpected finding tends to support the recent concept that some persons with fatty livers may indeed be “good fat storers” [59] who can sequester excessive lipid fuel as relatively benign triglycerides. Consistent with LAP’s relation to type 2 diabetes and similar clinical states [11]–[16], it has been proposed that triglyceride storage in liver tissue might be a marker of hepatic insulin resistance and diabetes risk, but these adverse effects of neutral triglyceride storage could be balanced by the protection it provides against lipotoxic damage to hepatocytes caused by some non-triglyceride fatty acids and their derivatives [59], [60]. In other non-adipose (ectopic) tissues there could be distinct roles for lipid storage. The functional consequences of increased intramyocellular lipid in skeletal muscle may differ depending on factors related to body fat distribution, ancestral origin, or habitual physical activity [61], [62].

Our study has limitations. We measured adiposity at only one point in time, so our estimates could not account for changes in adiposity. Our models also lacked information about changes in diet, physical activity or co-morbidities that might well have modified the likelihood of mortality. In addition, our use of circumferences at the waist, hip, and thigh was limited to the NHANES’ specific anthropometric protocols. Other studies or clinical settings may employ different protocols for measuring the waist, hip, or thigh. Our analytic sample provided no genetic markers of ancestral admixture, and we included only persons who described themselves as non-Hispanic whites, non-Hispanic blacks, and Mexican-Americans. It is possible that persons of other ancestries might experience different mortality outcomes in relation to their baseline adiposity indicators. Our NHANES sample of persons with LAP values (restricted to fasting participants) underrepresented high-risk diabetic patients because insulin users were not asked to fast before their NHANES exam.

Despite these limitations, our identification of some differences in health risk associated with adiposity indicators may help to focus research questions for the future. Indeed, the concept of LAP emerged initially from an intention to estimate inexpensively how lipid metabolites were accumulated ectopically with increasing age [8]. Basic research may increasingly focus on variations in the qualitative aspects and limits of lipid storage and how these characteristics may vary between tissues, regional depots, and organs. There will be complementary interests in the metabolic alterations and functional losses (“lipotoxicity”) that occur when the benign accumulation limits are exceeded [63]. As new technologies describe the quantities and actions of specific fatty-acid derivatives in various anatomic locations, future epidemiologic studies may then clarify how specific regional depots of adipose tissue are related positively or negatively to lipotoxic consequences in the liver, skeletal muscle, pancreas, and other non-adipose tissues. These emerging insights should improve our ability to recognize and address health risks in population subgroups defined by sex, age, or other characteristics.

## Supporting Information

Appendix S1 **Population-based cross-tabulations of the 6 participating adiposity indicators displaying the concordances (in bold) of assignments to adiposity quartile 1, midrange, and quartile 4.**
(XLSX)
